# Hemistepsin A Inhibits Cell Proliferation and Induces G0/G1-Phase Arrest, Cellular Senescence and Apoptosis Via the AMPK and p53/p21 Signals in Human Hepatocellular Carcinoma

**DOI:** 10.3390/biom10050713

**Published:** 2020-05-04

**Authors:** Su Youn Baek, Ui Wook Hwang, Ho Young Suk, Young Woo Kim

**Affiliations:** 1 Institute for Phylogenomics and Evolution and Institute for Korean Herb-Bio Convergence Promotion, Kyungpook National University, Daegu 41566, Korea; uwhwang1@gmail.com; 2 Department of Biology Education, Teachers College, Kyungpook National University, Daegu 41566, Korea; 3 Department of Life Sciences, Yeungnam University, 280 Daehak-ro, Gyeongsan 38541, Gyeongsangbuk-do, Korea; hsuk@yu.ac.kr; 4 School of Korean Medicine, Dongguk University, Gyeongju 38066, Korea

**Keywords:** hemistepsin A (HsA), hepatocellular carcinoma (HCC), AMP-activated protein kinase (AMPK/mTOR), p53, G0/G1 cell cycle arrest, apoptosis

## Abstract

Hemistepsin A (HsA), a natural sesquiterpene lactone isolated from *Hemistepta lyrata*, has been known as a wide range of anti-tumor effects. The aim of this study was to determine whether HsA suppresses hepatocellular carcinoma (HCC) and to figure out the cellular signaling pathways involved in the anti-HCC activities by experiments using the Huh7 cells (a human HCC cell line) and a xenograft HCC model. In this study, HsA completely inhibited HCC cell proliferation, presumably because it induced G0/G1 cell cycle arrest and mitochondrial-related apoptosis. HsA up-regulated p53, p21, cleaved caspase-3 and cleaved PARP (poly (ADP-ribose) polymerase), but reduced cyclin D, CDK6 and Bcl-2 expressions, and it disrupted mitochondrial membrane potential (ΔΨm). Moreover, phosphorylation of AMP-activated protein kinase (AMPK) was increased by HsA as did the resveratrol and 5-aminoimidazole-4-carboxamide ribonucleotide (AICAR, positive controls). Inhibition of AMPK by using compound C, a competent inhibitor of AMPK, attenuated the loss of ΔΨm, p53 up-regulation and cellular senescence. The efficacy of HsA to reduce HCC cell proliferation, compared to that of other known anti-HCC agents, appears to be similar or slightly better. The anti-tumor effect of HsA was also determined in mice, showing reduced growth of xenografted tumors with no weight loss. Overall, the results suggest that HsA should be considered as a candidate anti-HCC drug.

## 1. Introduction

Liver cancer was the second most fatal cancer of all cancer types in 2018 [1]. About 90% of malignant tumors in the liver are hepatocellular carcinoma (HCC), which mostly occurs in the presence of cirrhosis [2,3]. Although surgical methods, such as resection or transplantation, may be applied, if HCC is detected early, beyond the initial stage, chemotherapy is primarily used to stop tumor growth and reduce its size [4,5]. However, the clinical application of chemotherapy drugs should be undertaken with caution because of frequent drug-related side effects [4]. Therefore, it is necessary to discover new potent compounds that can minimize side effects, and in this sense, it is worth considering natural products. As a result of these needs and efforts, over 60% of current anti-tumor drugs have been derived from natural sources [6,7].

Sesquiterpene lactones (SQLs) are a class of sesquiterpenoid natural compounds containing a lactone ring that is often detected in plants of the Asteraceae family. It has been reported that various kinds of SQLs are involved in cancer cell cytotoxicity and anti-tumor efficacy [8,9,10], some of which have been regarded as useful in cancer clinical trials [11]. *Hemistepta lyrata* Bunge, an Asteraceae representative, has long been used to treat fever, hemorrhage and ulcers in traditional Korean medicine [12,13]. The components isolated from *H. lyrata* have been reported to inhibit the proliferation of cancer cells or induce apoptosis in a variety of cancer cell lines including lung, prostate and breast [14,15]. Although hemistepsin A (HsA) is an SQL isolated from *H. lyrata,* no study has been performed to determine whether HsA has an effect on HCC.

Most cancer chemotherapeutic strategies focus on the regulation of the cell cycle and the induction of apoptosis [16]. In general, apoptosis of cells occurs via the arrest of the cell cycle, in which several regulatory proteins are involved. It has been reported that p53 did not function properly in over 50% of the human cancer cases examined [17]. p53 activates the DNA repair proteins when the DNA is damaged, arrests the cell cycle at the G1/S point upon DNA damage recognition and initiates apoptosis if the DNA damage is not recoverable [18]. For these reasons, p53 is recognized as a so-called anti-tumor protein. Another well-known tumor suppression protein, p21, is expressed with the help of p53 and Miz-1 to inhibit cyclin-dependent kinases and stop the cell cycle [18]. Recent articles have demonstrated the functions of various proteins which induce p53-dependent metabolic checkpoints, typically AMP-activated protein kinase (AMPK) [19,20]. Activation of the AMPK pathway results in cross-talks with oxidative or genotoxic stresses regulating cell proliferation, which appears to be mediated via the Ras/PI3K/mTOR pathway and up-regulation of p53 and p21 [21,22]. The AMPK activation can thus be considered as a logical therapeutic target for the suppression of tumor cell proliferation [21].

In this study, we performed a series of tests to determine whether HsA contains a therapeutic effect against HCC and how it works in cellular and molecular signaling systems. In our results, HsA clearly induced G0/G1 cell cycle arrest and apoptosis, which were mediated by the regulation of the p53/p21 axis and the AMPK pathway. Our data also showed that HsA effectively suppressed the growth of Huh7 cells xenografted in nude mice. Our findings provide mechanistic insights into the therapeutic application of HsA in HCC-targeted clinics.

## 2. Materials and Methods

### 2.1. Chemicals and Reagents

Anti-PARP, anti-caspase 3, anti-ACC, anti-p-AMPK, anti-AMPK, anti-p-mTOR, anti-mTOR, anti-p-P70S6K1, anti-P70S6K1, anti-cyclin D1, anti-cyclin E1, anti-p53 and ant-β actin antibodies were purchased from Cell Signaling Technologies (Danvers, MA, USA). Anti-CDK2, anti-CDK6, anti-Bcl_2_, anti-BAX, and p21 antibody were purchased from Santa Cruz Biotechnology (Santa Cruz, CA, USA). Horseradish peroxidase (HRP)-conjugated goat anti-rabbit and goat anti-mouse IgGs were purchased from Zymed Laboratories (San Francisco, CA, USA). The compound C was purchased from Calbiochem (San Diego, CA, USA). The 3-(4,5-dimethylthiazol-2-yl)-2,5-diphenyl-tetrazolium bromide (MTT), propidium iodide (PI), JC-1 and all other reagents were purchased from Sigma-Aldrich (St. Louis, MO, USA). The HsA was kindly provided by Prof. Jong Rok, Lee (Daegu Haany University, Gyeongsan, Korea), and isolated as previously described [23]. The pure HsA (1.31 g) was isolated from the chloroform extract (150 g) of *H. lyrata,* and was stored at −20 °C. The purity (>97%) was verified by comparing retention time with standard compounds.

### 2.2. Cell Culture

Huh-7 cells, a human HCC cell line, were purchased from the Health Science Research Resources Bank (Osaka, Japan). The HepG2 and SK-Hep1 were obtained from the American Type Culture Collection (ATCC, Manassas, VA, USA), and SNU475 was purchased from the Korean Cell Line Bank (Seoul, Korea). The Huh7 cells were cultured in Roswell Park Memorial Institute Media 1640 (RPMI-1640), whereas HepG2, SK-Hep1 and SNU475 were cultured in Dulbecco’s modified Eagle’s medium (DMEM) with 10% fetal bovine serum (FBS). Dimethyl sulfoxide (DMSO) (the dilution rate = 1:1000) was used as vehicle. All of the control was treated with DMSO alone.

### 2.3. Cell Proliferation Assay

Cell proliferation and viability were assessed by MTT assay [24]. In metabolically active cells, MTT salt is cleaved by the action mitochondrial dehydrogenases and is reduced to an insoluble formazan crystal displaying a purple color that is detectable in spectrophotometer [24]. Cells were plated at a density of 1 × 10^5^ cells per well in 48-well plates and incubated for 24 h, which was followed by the incubation in medium containing various concentrations (from 5 to 10 μM) of HsA for 24 h.

### 2.4. Cell Cycle Analysis by Flow Cytometry

Huh7 cells were seeded in six-well plates at a density of 5 × 10^5^ cells [24]. On the following day, cells were treated with HsA at different concentrations (from 1 to 10 μM) for 24 h. After treatment and incubation, the cells were harvested and washing with phosphate-buffered saline (PBS). Cells were stained with BD Cycletest^TM^ Plus DNA kit (BD Biosciences, San Jose, CA, USA) according to the manufacturer’s instruction, and analyzed on the Accuri C6 (BD Biosciences, Mountain View, CA, USA). All experiments were performed in triplicate.

### 2.5. Senescence-Associated β-Galactosidase (SA-β-gal) Staining

The Senescence β-Galactosidase Staining Kit (Cell Signaling Technology, Danvers, MA, USA) was used to evaluate β-galactosidase activity, a known characteristic of senescence, in Huh7 cells according to the manufacturer’s instructions. Images were taken using phase-contrast microscopy (Olympus, Tokyo, Japan).

### 2.6. Apoptosis Analysis by Flow Cytometry

Cells were seeded in six-well plates at a density of 5 × 10^5^ and incubated for 24 h. On the following day, HsA was treated at different concentrations, ranging from 5 to 25 μM and incubated for 24 h. After HsA treatment and incubation, cells were harvested, washed with cold PBS and resuspended in the 1× binding buffer. The cells were incubated with FITC-conjugated Annexin V and PI for 15 min in the dark at room temperature, and were analyzed on the Accuri C6 based on the previously described protocol [25].

### 2.7. Mitochondrial Membrane Potential (ΔΨm) Analysis

Change in the mitochondrial transmembrane potential (ΔΨm) was measured using JC-1, a fluorescent carbocyanine dye based on the previously described protocol [24]. The JC-1 spontaneously forms complexes known as J-aggregates with red fluorescence in physiologically healthy cells with sufficient mitochondrial ΔΨm, whereas it remains in the monomeric form, which exhibits only green fluorescence, in apoptotic or unhealthy cells with low ΔΨm [26]. Huh7 (5 × 10^5^ cells) were cultured in a 6-well plate for 24 h and were treated with different concentrations of HsA (0, 5, 10 and 25 μM) for 24 h with or without compound C (10 μM). After treatment, cells were incubated with 10 μM JC-1 in media for 30 min in the dark and later collected. After washed with PBS, cells were subjected to flow cytometry analysis to measure the ratio of JC-1 aggregate (red) to monomer (green) based on the color intensity.

### 2.8. Western Blotting Analysis

The preparation of cell lysates and the process of Western blot analysis were performed as previously described [24]. The protein bands were detected using the Fusion Solo scanning system (Vilber Lourmat, Paris, France). Each band intensity was estimated by the calculation of the ratio to the intensity of each actin band, which was implemented by Image J software (https://imagej.nih.gov/ij/download.html). Antibodies’ information is summarized in Supplementary Table S1.

### 2.9. Wound Healing Migration Assay

Huh7 cells were plated on 6-well plates at 90% confluence. The monolayer was wounded using a 200 μL tip. After wounding, detached cells were further incubated with or without various concentrations of HsA. After 0, 24, and 48 h, these cells were rinsed with serum-free medium and fixed in absolute methanol. Cell migratory behavior was then observed under phase-contrast microscopy (Olympus) and recorded.

### 2.10. Xenograft Mouse Model

All animal experimental procedures were approved by the Yeungnam University Institutional Animal Care and Use Committee (Protocol Number 20180203) and were conducted in accordance with the guidelines of the National Institutes of Health. The BALB/c-nu mice (5–6 weeks old) were purchased from Charles River Orient Bio (Seongnam, Korea). The Huh7 cells (1 × 10^7^) were inoculated subcutaneously on the back of nude mice and allowed to grow for 10 days to reach a tumor volume of approximately 100 mm^3^. The mice were randomly divided into three groups; individuals were orally administered by either HsA (5 or 10 mg/kg dissolved in water) or vehicle (only water) every three days for nine days. The level of tumor growth was monitored by measuring the size every 3 days using a digital caliper. The tumor volume was quantified using the following formula: 0.5 × *a* × *b*^2^, where “*a*” refers to the long diameter and “*b*” to the short diameter of the tumor. Body weight was also checked every three days throughout the treatment period.

### 2.11. Statistical Analysis

Statistical analysis was performed using the GraphPad Prism software version 5.01 (Graph Pad Software, La Jolla, CA, USA). Student’s *t*-test and one-way analysis of variance (ANOVA) were used for testing whether significant differences existed among the groups. Statistical significance was regarded if *p* < 0.05. All data were described as mean ± SD quantified from repeated independent experiments.

## 3. Results

### 3.1. HsA Reduces Proliferation of Hepatocellular Carcinoma Cells

**Figure 1:**
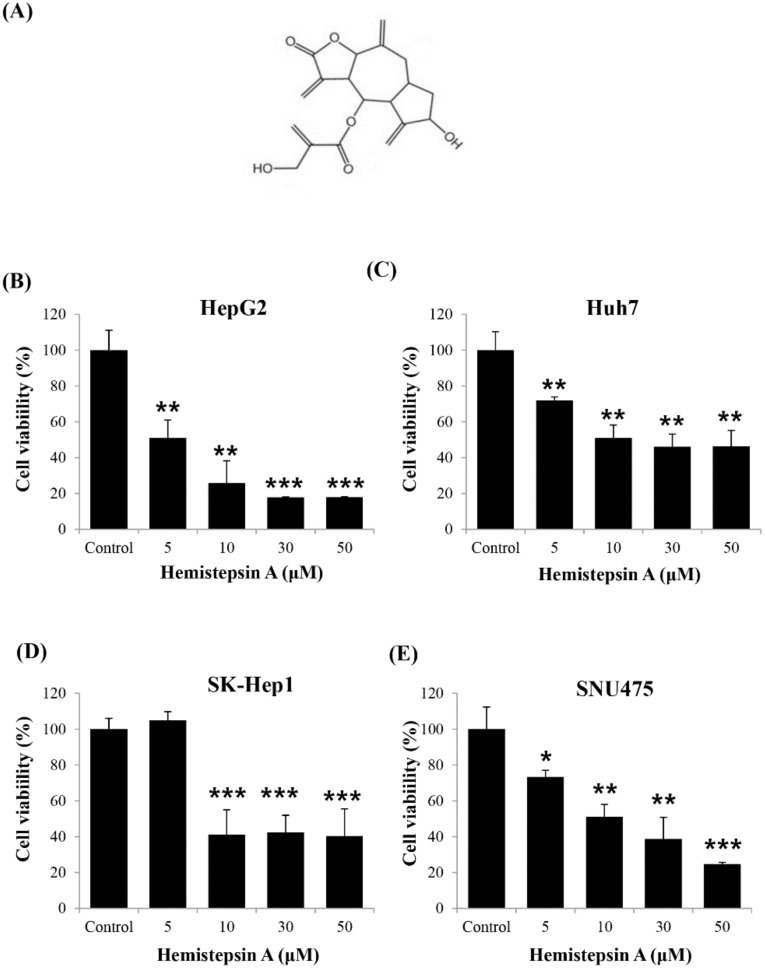
Effect of hemistepsin A (HsA) on the proliferation of human hepatocellular carcinoma (HCC) cells. (**A**) The chemical structure of HsA. The dose-dependent inhibitory effects of HsA on the proliferation of different cell lines, HepG2 (**B**), Huh7 (**C**), SK-Hep1 (**D**) and SNU475 cells (**E**), were assessed using MTT assay (*n* = 3). Results were described as the percentages of cell proliferation in the cell lines treated with HsA relative to those in the vehicle-treated controls. Values were described as the mean ± SD measured from three wells per treatment. * *p* < 0.05; ** *p* < 0.01; *** *p* < 0.001.

To determine whether HsA has an anti-tumor effect, we investigated whether it affected cell viability in various human HCC cell lines by using the MTT assay. Four human HCC cell lines, HepG2, SK-Hep1, SNU475 and Huh7, were treated with HsA at different concentrations (0, 5, 10, 30 and 50 μM) for 24 h. The results showed the higher the concentration of HsA, the lower the viability of the cells. The proliferation of carcinoma cells was significantly inhibited at HsA concentrations above 10 μM. (Figure 1B–E). Moreover, the IC_50_ of HsA (the concentration of an inhibitor where the response is reduced by half) was calculated to predict the survival rate of each cell line. Among the cell lines, both HepG2 and Huh7 appeared to be sensitive to HsA treatment (Supplementary Table S2). However, Huh7 cells were used in our subsequent experiments, since HepG2 are known weakly to be tumorigenic in nude mice that are used in our in vivo tests [27].

### 3.2. HsA Induces G0/G1 Cell Cycle Arrest and Cellular Senescence in Huh7 Cells

**Figure 2:**
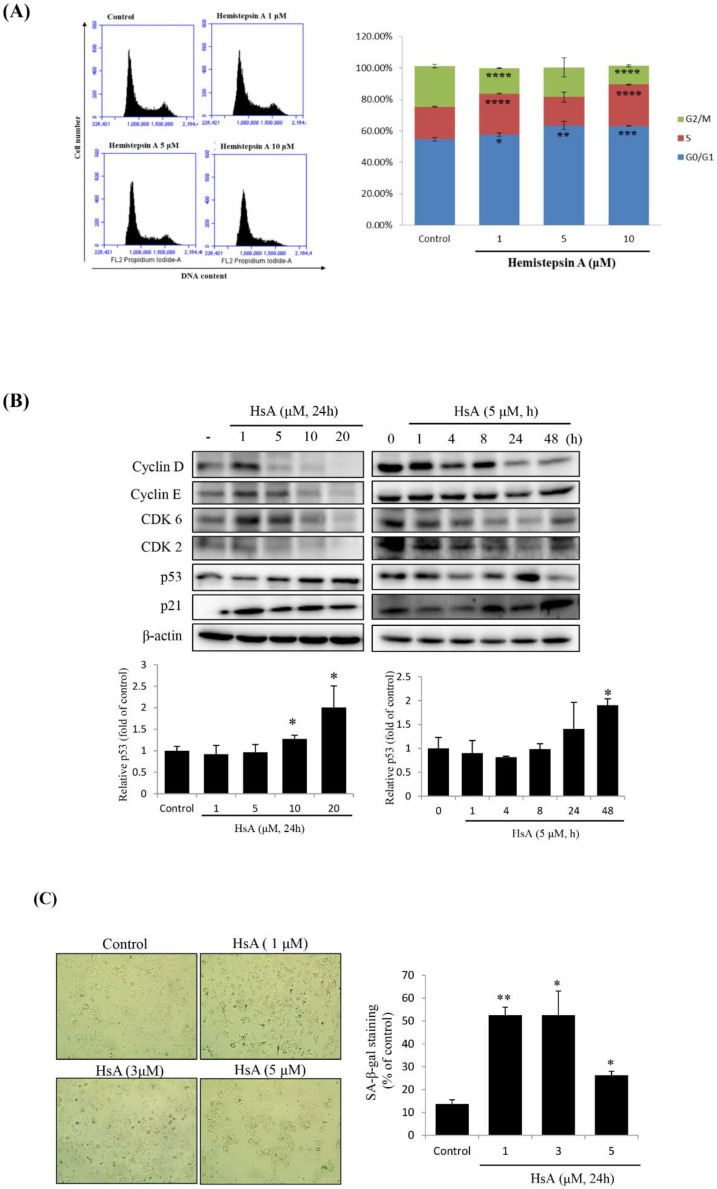
HsA-induced G0/G1 cell cycle arrest and cellular senescence in Huh7 cells. Huh7 cells were treated with either various concentrations of HsA (0, 1, 5, 10, 20 μM) for 24 h or for various times (0, 1, 4, 8, 16 or 24 h) with 5 μM HsA. (**A**) Cell cycle profiles were measured using flow cytometry following the treatments of the cells with various concentrations of HsA for 24 h (*n* = 3). This test was not performed at 20 μM. (**B**) Western blotting was performed to examine the dose- (left) and time-dependent (right) change in the cellular level of cyclin D, cyclin E, CDK2, CDK6, p53 and p21 with the treatment of HsA. β-actin was used for loading control. Protein levels of p53 were presented as relative band intensities to the control (vehicle-treated) group (the lower panel). (**C**) Cellular senescence was examined by staining of senescence-associated β-galactosidase (SA-β-gal). Senescent cells (blue-stained) were observed by bright-field microscopy, and the percentage of SA-β-gal-positive cells was quantified for every treatment group (0, 1, 3 and 5 μM). Data were described as the mean ± SD obtained from three separate experiments. * *p* < 0.05; ** *p* < 0.01; *** *p* < 0.001; **** *p* < 0.0001.

To determine the cellular mechanism that has prevented the proliferation of HCC cell lines, we examined the cell cycle profiles in Huh7 cells treated with HsA at various concentrations or times by using flow cytometry. When the cells were treated with HsA, the proportion of G0/G1 phase cells was relatively high, but the proportion at the G2/M phase was relatively low, compared with the cell phase distributions in the control cells. The proportion of the G0/G1 phase tended to remarkably increase by treatment of 5 μM HsA and above (Figure 2A and Supplementary Figure S1). In addition, HsA down-regulated the expression levels of checkpoint proteins involved in the regulation of the G1/S transition including cyclin D1, cyclin E and CDK6, but up-regulated the expression levels of p21 and p53 in both dose- and time-dependent manners (Figure 2B).

Since p53 and p21 have important roles in cellular senescence through their mediation of G1-phase cell cycle arrest [24], the SA-β-gal staining technique was used to define HsA-induced cellular senescence. We observed the signature of HsA-induced cellular senescence, although maximum senescence was observed at a relatively low HsA concentration (1 μM; Figure 2C). Normally, senescence occurs before apoptosis, so low concentrations should be used. These results indicated that HsA could activate the p53-p21 pathways, probably leading to the observed cell cycle arrest at the G0/G1 phase and finally the cellular senescence.

### 3.3. HsA Induces Apoptosis and Mitochondrial Dysfunction in Huh7 Cells

**Figure 3:**
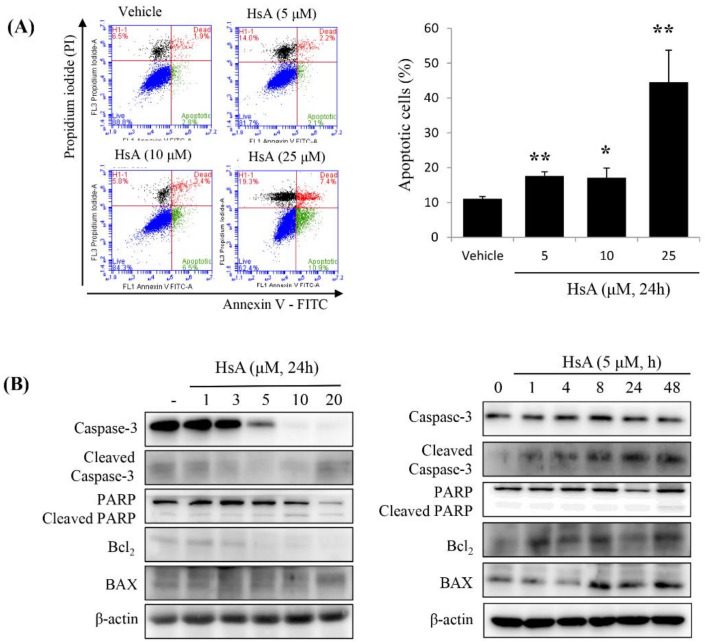

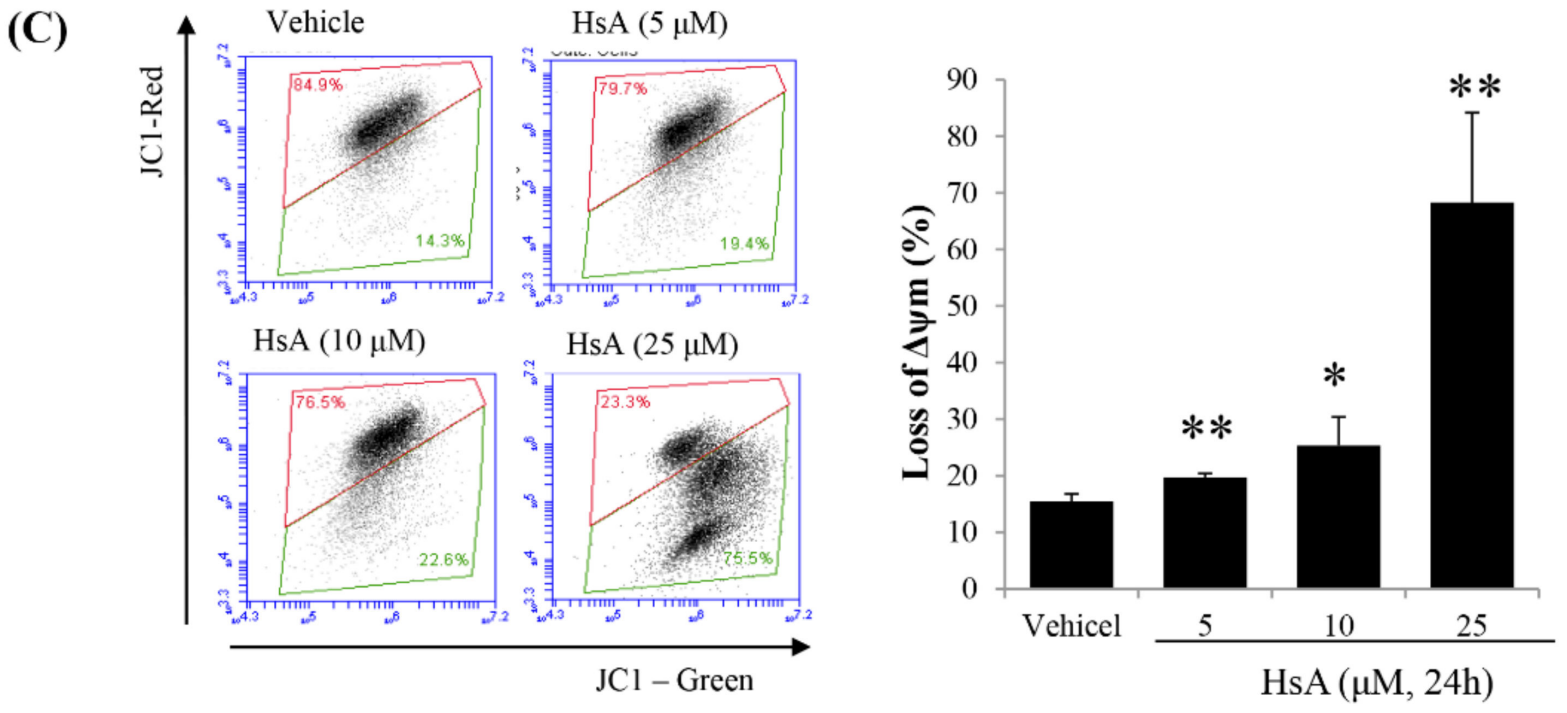
HsA induces apoptosis via the mitochondrial pathway in Huh7 cells. (**A**) The results of flow cytometric analysis showing HsA-induced apoptosis in Huh7 cells based on Annexin V-FITC/PI staining (left). Data in the lower right (Annexin V+/PI−; green), in the upper right (Annexin V+/PI+; red) and in the upper left quadrant (Annexin V-/PI+; black) represent the states of early apoptotic, late apoptotic and necrotic cells, respectively. The apoptotic level difference in response to increasing HsA concentrations is visualized in the graph (right). (**B**) The results of Western blotting analysis showing the change of apoptotic protein expression level in response to increasing HsA concentrations (left) and increasing time at an equal concentration (right). β-actin was used as a loading control. (**C**) Decrease in mitochondrial potential (ΔΨm) induced in response to increasing HsA concentrations. After treatment with HsA, the cells were stained with JC-1 for 30 min and analyzed by flow cytometry. Changes in ΔΨm were detected by the ratio of red fluorescence (JC-1 aggregates) to green fluorescence (JC-1 monomers; left; *n* = 3) and the data were statistically compared (right). Data were represented as the mean ± SD obtained from three separate experiments. * *p* < 0.05; ** *p* < 0.01.

We then examined whether HsA could induce apoptosis of Huh7 cells. The apoptotic rates were measured using flow cytometry. Apoptotic cells, including early and late apoptotic cells (annexin V-positive and PI-negative or PI-positive), were induced by treatment with 5 μM HsA (annexin V/PI ratio, 18.2%), and their numbers increased in a dose-dependent manner, though a similar apoptotic response was observed at 5 μM and 10 μM (Figure 3A). To further characterize the cell death process, we investigated the expressions of downstream apoptotic proteins by Western blot assays. HsA increased the level of poly-ADP-ribose polymerase (PARP) cleavage and caspase-3 cleavage but decreased the level of Bcl_2_ in Huh7 cells (Figure 3B).

Since early apoptosis is accompanied by the damage of mitochondrial membrane [28], we measured the effect of HsA on the alternation of mitochondrial membrane potential (ΔΨm). In Huh7 cells treated with various doses of HsA for 24 h, green fluorescence intensity was high whereas red fluorescence intensity decreased in a dose-dependent manner, indicating mitochondrial dysfunction (Figure 3C). These results suggest that intrinsic apoptosis is induced following the addition of HsA via mitochondrial dysfunction.

### 3.4. HsA Reduces the Proliferation of HCC Cells by Activating AMPK Signaling

**Figure 4:**
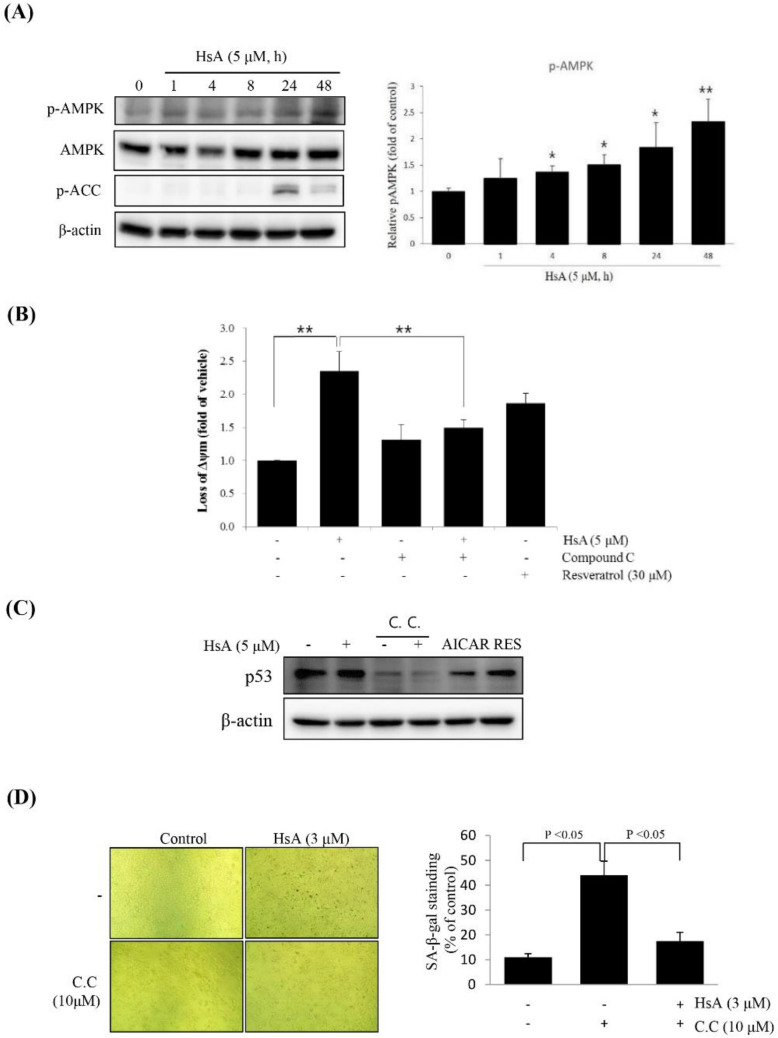
HsA inhibited cell proliferation through AMP-activated protein kinase (AMPK) in Huh7 cells. (**A**) HsA-induced phosphorylation of the proteins associated with the AMPK pathway. Western blot analyses were performed with the lysates of cells that had been treated with 5 μM HsA for the indicated time period (right). The band intensities at different concentrations of HsA were visualized and statistically compared (right). Beta-actin served as a loading control. (**B**) HsA-induced loss of ΔΨm was revered by treatment with 10 μM C.C for 1 h. The ΔΨm was evaluated with JC-1 stain by flow cytometry (*n* = 3; upper). The data were visualized and compared statistically (lower). (**C**) Inhibition of AMPK activation with C.C reduced HsA-induced p53 expression. Western blot analysis of p53 was performed with lysates of Huh7 cells that had been pretreated with 10 μM C.C for 1 h being followed by exposure to 3 μM HsA for 24 h. (**D**) SA-β-gal staining was performed, and the SA-β-gal-positive cells were quantified (*n* = 4; upper) and the data were statistically compared (lower). Data were represented as the mean ± SD for four replicates. * *p* < 0.05; ** *p* < 0.01.

To identify the molecular signal transduction, which is responsible for the effect of HsA, we analyzed the expression of AMPK and related downstream proteins. HsA treatment promoted the phosphorylation of AMPK in a time-dependent manner (Figure 4A). 

To determine whether the effect of HsA was related to the AMPK, we stimulated Huh7 cells with compound C, a competitive inhibitor of AMPK. In the co-treatment of compound C with HsA, the HsA-induced loss of ΔΨm was significantly suppressed in Huh7 cells (Figure 4B). Furthermore, compound C reduced the level of HsA-stimulated SA-β-gal-positive cells and obviously inhibited the expression of p53 (Figure 4C,D). These results suggest that HsA activates AMPK and p53 to inhibit the malignant properties of HCC cells.

### 3.5. HsA Inhibits Tumor Migration, Growth and Proliferation

**Figure 5:**
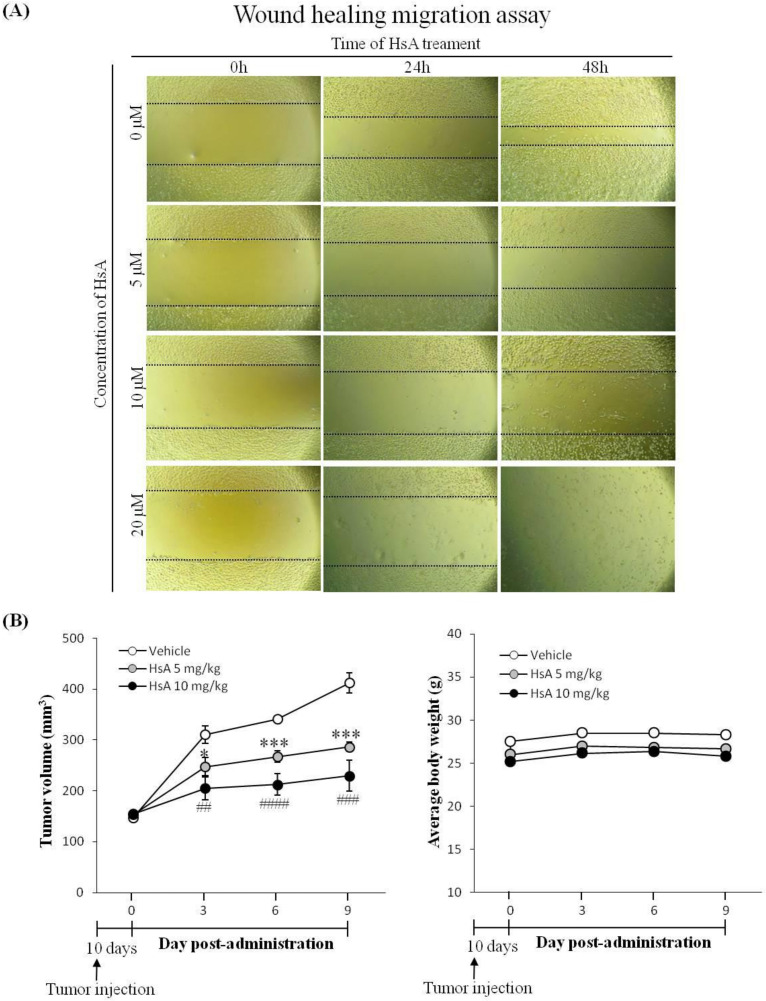
HsA inhibits cell migration and tumor growth in animals. (**A**) A wound healing migration assay was used to investigate the motility of Huh7 cells. Original magnification ×200. (**B**) After tumor development, mice were orally treated once per three days with 5 mg/kg, or 10 mg/kg of HsA, or saline. Tumor volume (left) and body weight (right) were measured (*n* = 3).

Cancer cell migration is a key property of tumor growth, angiogenesis and metastasis [29]. To examine the effect of HsA in vitro, wound healing migration assays were performed. HsA was found to effectively inhibit the migration of Huh7 cells induced with FBS in a dose-dependent manner (Figure 5A). The cell-free area of the HsA treatment group was wider than that of the control group in both dose- and time-dependent manners (Figure 5A). These data indicate that HsA effectively inhibits the malignant properties of HCC cells.

To evaluate the anti-tumor effect of HsA in vivo, a xenograft HCC model was established by subcutaneous inoculation of Huh7 cells to the flanks of nude mice. At a certain time after the inoculation of Huh7 cells, obvious lumps were visible under the skin at the inoculation site. The Huh7 inoculation did not change mouse behavioral activities such as eating, drinking and excretion during the experiment. After the tumor volume reached 100 mm^3^, mice were orally administered HsA (0, 5 and 10 mg/kg) for nine days. HsA suppressed the growth of the tumors in a dose-dependent manner (Figure 5B). However, no significant difference was found between the average weights of the groups, suggesting that HsA produced no side effects that could threaten the health of the HCC-bearing nude mice.

**Figure 6:**
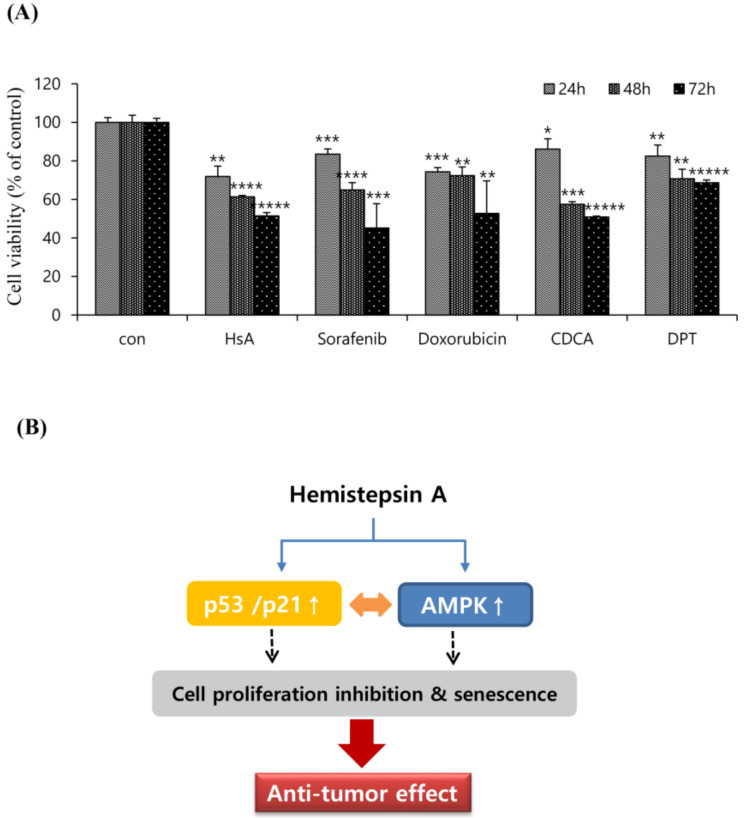
Compared to well-known drugs to HCC, HsA effectively inhibited malignant properties. (**A**) Huh7 cells were treated with test agents at the indicated concentrations. All data were calculated after 24, 48, and 72 h of post-treatment (*n* = 3). Results were described as the percentages of cell proliferation in the cell lines treated with agents relative to those in the vehicle-treated controls. Data represent the mean ± SD from three separate experiments. * *p* < 0.05; ** *p* < 0.01; *** *p* < 0.001; **** *p* < 0.0001; ***** *p* < 0.0001. (**B**) Schematic representation of HsA-induced cytotoxicity in Huh7 cells. HsA-induced AMPK phosphorylation, and elevated the levels of p53 and p21, which contributed to the cell cycle arrest and cell proliferation inhibition.

To examine the relative anti-tumor effectiveness of HsA, we analyzed the antiproliferative activity of HsA and other agents effective in the treatment of HCC that have been reported in a variety of biological studies. The effects of HsA (7 μM), sorafenib (10 μM), doxorubicin (1.0 μM), chenodeoxycholic acid (CDCA; 150 μM) and deoxypodophyllotoxin (DPT; 100 μM) on Huh7 cells viability were investigated for three different incubation times using the MTT assay (Figure 6A). The inhibition rates of these agents on Huh7 cells were significantly different (*p*  <  0.05) from each other, and the incubation time significantly affected cell viability (*p*  <  0.05). As a result, HsA has been proved to have an efficacy comparable to other natural products or commercially available medical reagents that have been known to treat liver cancer.

## 4. Discussion

Various natural compounds have been shown to stop cancer cell growth by arresting the cell cycle [25,30]. Sesquiterpene lactones (SQLs) are one of the representative medicinal substances derived from many natural sources. Many SQLs have been reported to exhibit anti-tumor efficacy, some of which have already been applied in clinical practice [11]. However, SQLs cannot always be expected to have favorable effects, given the possibility of an SQL showing toxicity or non-selectivity depending on its molecular structure or cancer type [31].

In this study, we observed that HsA can mediate G0/G1 cell cycle arrest directly, leading to the inhibition of proliferation in Huh7 cells. HsA effectively down-regulated the expression of checkpoint proteins regulating the G1/S transition including cyclin D1, and CDK6, whereas it up-regulated p21 and p53. The p53 has been implicated to be one of the critical protein components in cell senescence [32]. Senescence is an irreversible cell cycle arrest that can be considered a robust physiological anti-tumor response. Therefore, innovative therapeutic approaches can be designed to induce tumor cell regression by re-activating senescence-signaling proteins [32]. In our results, low doses of HsA enhanced the level of SA-β-gal staining in Huh7 cells, which is indicative of p53 pathway-mediated senescence.

Apoptosis signaling cascades are divided into two major pathways, the death-receptor and mitochondria-mediated pathway [33]. In this study, we demonstrated that HsA treatment reduced the expression of Bcl-2, an important regulator in the mitochondrial-mediated apoptosis pathway. In addition, HsA significantly increased the protein levels of cleaved caspase-3 and PARP, while it decreased ΔΨm in a dose-dependent manner. These results indicate that HsA-induced apoptosis occurs through mitochondrial damage.

Mitochondrial dysfunction is known to inhibit glycolysis and ATP production, which can result in the accumulation of cellular AMP and the activation of AMPK [34]. The AMPK activation has been reported to suppress the proliferation of various cancers via the regulation of cell cycle progression, apoptosis, autophagy, inhibition of protein synthesis and de novo fatty acid synthesis [35]. Indeed, decreased AMPK activity in patient tumors is reported to be associated with aggressive clinical phenotype or poor prognosis [36]. In previous studies, the knockdown of AMPK resulted in greater tumorigenicity, and AMPK activation is associated with inhibition of HCC by cell senescence and autophagy [37,38,39]. Our results showed that HsA up-regulated AMPK phosphorylation, but attenuated HCC cell growth and viability. Moreover, the inhibition of AMPK activity in Huh7 cells by compound C reduced HsA-induced ΔΨm loss, indicating a relationship between HsA-induced AMPK activation and HCC cell apoptosis. Recently, researchers have speculated that the AMPK/mTOR signaling pathways are the main axes in regulating the proliferation and survival of cancer cells [40]. Acting via TSC2 and AMPK, directly and indirectly, suppress mTOR activity. We thus speculate that the treatment effect of HsA is associated with the activation of the AMPK/mTOR axis, and that AMPK may be one of the pathways involved in HsA-induced cytotoxicity (Figure 6B).

It has been shown that AMPK activation leads to the up-regulation and phosphorylation of p53 at Ser15 [22]. Previously, the checkpoint in the cells was also down-regulated in the p53-deficient mouse embryonic fibroblasts (MEF) cells [22]. Metformin, another activator of AMPK, can inhibit the growth of esophageal squamous cell carcinomas in both cultured cells and animal models through the involvement of p53, p21, p27 and cyclinD1 [38]. In this study, we showed that compound C treatment can inhibit HsA-induced cellular senescence as well as HsA-induced expression of p53. In our nude mice system xenografted with Huh7 cells, HsA reduced tumor growth without body weight loss or any visible health problems.

Our results revealed that HsA has comprehensive therapeutic capabilities. Previous studies have shown that HsA has potential anti-cancer and anti-inflammatory properties, and it has also been found that the anti-inflammatory properties were excellent in the liver [15,41]. A recent study showed that HsA can effectively inhibit liver fibrosis [41]. Among the therapeutic functions of HsA, untested was the role in HCC, but our research revealed its potential for the effective treatment of HCC. The industrial value and effectiveness of HsA can be highly appreciated in that it is a natural medicine that works effectively from the onset of the disease to the malignant stage. What should be tested in future studies is whether HsA has a particularly comprehensive disease treatment capability only in liver tissue, or it is similar in other tissues. Moreover, since this study was conducted as part of a long-term project for the discovery and comparison of therapeutic substances in HCC induced by hepatitis C whose vaccine has not been yet developed, a HCV background cell line (i.e., Huh7) was used as the main cell line from a forward-looking perspective. This is another reason to choose the Huh7 cell lines.

## 5. Conclusions

The HsA suppressed the growth of the HCC in vitro model through activating AMPK, enhancing p53 activity and consequently inducing G0/G1 cell cycle arrest and mitochondrial-mediated apoptosis. HsA significantly inhibited migration and growth of tumor cells in vivo, without any obvious toxicity. However, it is required to clarify what iares the specific downstream molecular targets of HsA as well as which signaling is involved. This study provided critical insight into the underlying cellular and molecular mechanisms involved in HsA’s effects on HCC, and created a variety of natural, low-side-effect and low-cost options that could help with or after the treatment of HCC.

## Supplementary Materials

The following are available online at https://www.mdpi.com/2218-273X/10/5/713/s1, Figure S1: Cell cycle profiles, Table S1: The information of antibodies, Table S2: The half-maximal inhibitory concentration of HsA.
