# Supplementary material for: Hemistepsin A Inhibits Cell Proliferation and Induces G0/G1-Phase Arrest, Cellular Senescence and Apoptosis Via the AMPK and p53/p21 Signals in Human Hepatocellular Carcinoma
Source: Biomolecules. 2020 May 4;10(5):713. doi: 10.3390/biom10050713 (PMC7277854; doi:10.3390/biom10050713)
Supplement: Supplementary file 1 [file biomolecules-10-00713-s001.pdf]

Supplementary Fig. 1. Cell cycle profiles were measured using flow cytometry following the treatments of the cells with various concentrations of HsA for 24h. A total of 10,000 cells (within the red boundaries) were selected per each treatment. X axis indicates FSC (cell volume), and Y axis indicates SSC (cell granularity).

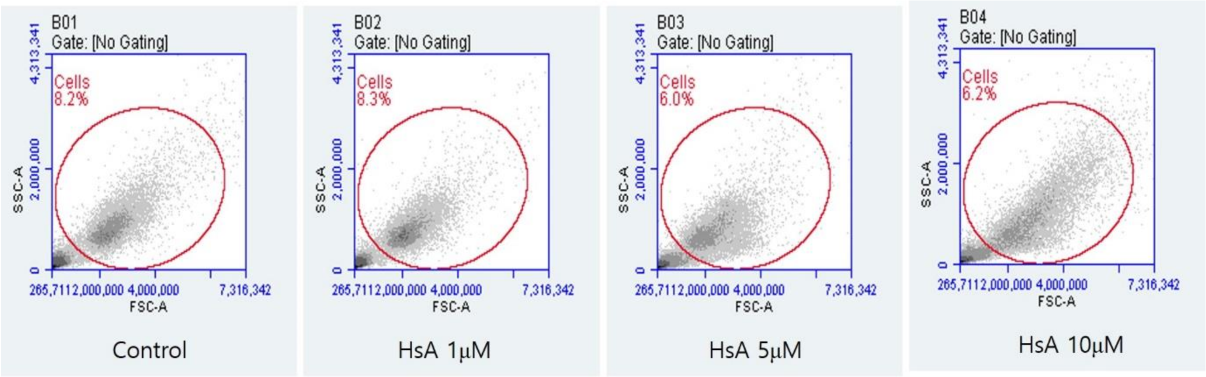

Supplementary Table 1. The information of antibodies used in this study. Data comprise manufacturer, item number and dilution condition.

Antibody name	Host	Manufacturer	Item number	Dilution ratio
Anti-PARP	Rabbit	Cell Signaling Technologie	9542	1:1000
anti-caspase3	Rabbit	Cell Signaling Technologie	9662	1:1000
anti-ACC	Rabbit	Cell Signaling Technologie	3661	1:1000
anti-p-AMPK	Rabbit	Cell Signaling Technologie	2531	1:1000
anti-p-mTOR	Rabbit	Cell Signaling Technologie	2971	1:1000
anti-mTOR	Rabbit	Cell Signaling Technologie	2972	1:1000
anti-p-P70S6K1	Rabbit	Cell Signaling Technologie	9205	1:1000
anti-cyclin D1	Rabbit	Cell Signaling Technologie	2978	1:1000
anti-cyclin E1	mouse	Cell Signaling Technologie	4129	1:1000
anti-p53	Rabbit	Cell Signaling Technologie	9282	1:1000
anti-CDK2	mouse	Santa Cruz Biotechnology	sc-6248	1:500
anti-CDK6	mouse	Santa Cruz Biotechnology	sc-7961	1:500
anti-Bcl2	mouse	Santa Cruz Biotechnology	sc-7382	1:500
anti-BAX	mouse	Santa Cruz Biotechnology	sc-7480	1:500
anti-p21	mouse	Santa Cruz Biotechnology	sc-6246	1:500
Goat anti-Rabbit IgG (H+L) Secondary Antibody, HRP	Goat	Zymed Laboratories	65-6120	1:5000
Goat anti-Mouse IgG (H+L) Secondary Antibody, HRP	Goat	Zymed Laboratories	65-6520	1:5000

Supplementary Table 2. The half-maximal inhibitory concentration (IC_{50}) of HsA on several hepatocellular cacinoma cells.

**Cell lines**	**IC_{50} (μM)**
HepG2	4.792
SK-Hep1	7.89
SNU475	5.12
Huh7	4.93
